# First discovery of the genus *Himalopenetretus* (Coleoptera, Carabidae, Patrobini) in China, with description of a new species

**DOI:** 10.3897/zookeys.997.58125

**Published:** 2020-11-25

**Authors:** Weifeng Yan, Hongliang Shi, Hongbin Liang

**Affiliations:** 1 College of Forestry, Beijing Forestry University, Beijing 100083, China College of Forestry, Beijing Forestry University Beijing China; 2 Key Laboratory of Zoological Systematics and Evolution, Institute of Zoology, Chinese Academy of Sciences, Beijing 100101, China Institute of Zoology, Chinese Academy of Sciences Beijing China

**Keywords:** Himalaya range, new species, Patrobini, Tibet

## Abstract

The Western Himalayan genus *Himalopenetretus* is firstly recorded from China, with one new species described, *H. burangensis***sp. nov.** (type locality: Burang county, Xizang).

## Introduction

*Himalopenetretus* Zamotajlov (Patrobini, Deltomerina) is a small genus previously containing only two species known from India and Pakistan (Löbl and Löbl 2017). This genus was erected for *H. franzi* (Zamotajlov & Sciaky, 1998) and *H. falciger* (Heinz & Ledoux, 1990), previously considered as belonging to the genus *Ledouxius*, based on the results of a phylogenetic analysis (Zamotajlov 2002). *Himalopenetretus* can be distinguished from the related genera mainly by the strongly elongated mandibles without an apical tooth.

In the summer of 2019, an expedition to Western Xizang was undertaken, during which a special specimen of Patrobini was collected in Burang County, near the border between China, Nepal and India. It was found under a rock in an alpine meadow near a glacier, together with several individuals of a small *Amara* species. The specimen was readily recognized as a new species of the genus *Himalopenetretus*. It proved to be most similar to *H. franzi*, distributed in Gangotri (North-West India), about 200 km west of the type locality of the new species. This is the first record of the genus *Himalopenetretus* from China. The main purpose of this paper is to record the genus from China and describe the new species; in addition, relationships between species of *Himalopenetretus* are briefly discussed.

## Materials and methods

The holotype and only examined specimen of the new species is deposited in the collections of the Institute of Zoology, Chinese Academy of Sciences, Beijing, China (**IZAS**). The methods of dissection, illustrations, and measurements mainly follow Shi et al. (2013).

Abbreviations of measurements used in the paper are as follows: L: overall length from apex mandibles to apex of elytra, measured along elytral suture; HW: width of head, as greatest transverse distance of head; PL: length of pronotum, as linear distance from anterior to basal margin, measured along the midline; PW: width of pronotum, as greatest transverse distance of pronotum; EL: length of elytra, as linear distance from basal ridge to apex, measured along elytral suture; EW: width of elytra, as greatest transverse distance of the two closed elytra.

## Taxonomy

### Himalopenetretus

Taxon classificationAnimaliaColeopteraCarabidae

Zamotajlov, 2002: 98

670F69B3-9C57-5BC9-A63A-11CBD693A271

#### Type species.

*Ledouxius franzi* Zamotajlov & Sciaky, 1998

#### Generic characters.

The genus can be identified by the following combination of character states: a medium sized Patrobini with dorsal side reddish brown; body elongate with inconspicuous eyes; first antennomere plurisetose, with one seta distinctly longer than the others; head with four to six pairs of setae between eyes and neck constriction; mandibles strongly elongated, apical tooth absent; submentum with two pairs of setae; lateral margins of pronotum with two to five pairs of setae before middle; elytra without scutellar pore, discal setiferous pores only present on the third interval; fifth meso- and metatarsomeres glabrous or with only a few very minute setae ventrally; apical lamella of aedeagus narrow, nearly straight in lateral view and more or less twisted leftwards in dorsal view, without a tooth or other apical protuberances; endophallus with two groups of copulatory pieces: proximal one near middle, with sharp and hooked apex and a rudimentary flagellum; distal one near end of apical orifice, smaller and bilobed.

*Himalopenetretus* is most similar to the genus *Ledouxius*, as follows: first antennomere plurisetose; submentum with two pairs of setae; lateral margins of pronotum plurisetose; fifth meso- and metatarsomeres without long setae ventrally. However, it can be differentiated from this genus by: (1) head with four to six pairs of setae between eyes and neck constriction; (2) mandibles strongly elongated; (3) eyes less prominent, pronotum subcordate to nearly quadrate; (4) elytra without scutellar pore; (5) apical lamella of aedeagus narrow and more or less twisted leftwards in dorsal view, endophallus possessing a rudimentary flagellum on proximal copulatory pieces. The subgenus Hasarotretus of the genus *Ledouxius* especially resembles *Himalopenetretus* in the absence of apical teeth on the mandibles, but is different in the many important characters mentioned above.

Besides with *Ledouxius*, species of *Himalopenetretus* also share some characters with the genus *Deltomerodes*, including the extremely long temporae, the leftwards twisted apical lamella of the aedeagus, and basal part of proximal copulatory piece forming a flagellum. However, the latter genus is different in: (1) mandibles normal, not elongated; (2) head with two to four pairs of setae between eyes and neck constriction; (3) pronotum somewhat elongate and flask-shaped, lateral margins with only one (rarely two) pairs of setae before middle; (4) elytra with additional discal setiferous pores present on the fifth or seventh intervals.

*Himalopenetretus* includes the following three species occurring in the Western Himalayas (Map 2):

*Himalopenetretus falciger* (Heinz & Ledoux, 1989): Pakistan (Gilgit);

*Himalopenetretus franzi* (Zamotajlov & Sciaky, 1998): India (Uttar Pradesh, Tapovan);

*Himalopenetretus burangensis* sp. nov.: China (Xizang, Burang).

### Himalopenetretus burangensis sp. nov.

Taxon classificationAnimaliaColeopteraCarabidae

6225D837-CC9B-5984-AC60-3F5BE00EE4C1

http://zoobank.org/1A44B1F4-43D7-4F6D-8FCF-F66249B06181

#### Type locality.

China, Xizang: Burang County.

#### Type material.

***Holotype***: male (IZAS), “Xizang, Ngari Pref., Burang County, Burang Town. N of Tinkar pass, alpine meadow. 30.2085N, 81.0661E, 4800m.”; “under stone, 2019. VII. 15, Shi HL lgt., Exp. BJFU 2019”; “DNA sample series number SHL2019--Xiz006”; “HOLOTYPE♂ *Himalopenetretus burangensis* sp. nov., des. YAN & SHI, 2020” [red label].

#### Diagnosis.

The new species is diagnosable in the genus by: tarsomeres dorsally glabrous; pronotum subquadrate, lateral margins straight before posterior angles, which are a little pointed, disc with a pair of small concavities to the side of the median line, lateral margins with two or three pairs of setae before middle; apical lamella of aedeagus elongate and evidently twisted leftwards, apex a little capitate and hooked leftwards.

The new species is most similar to *H. franzi*, but the latter species is different from it in: pronotum subcordate, lateral margins evidently sinuate before posterior angles, disc without concavities, lateral margins with four or five pairs of setae before middle; apical lamella of aedeagus shorter, only very weakly twisted leftwards, apex simple, narrowly triangular. The other species of the genus, *H. falciger*, is quite different from these two species in having tarsi dorsally pubescent and in many other respects.

#### Description.

**Figure 1.**
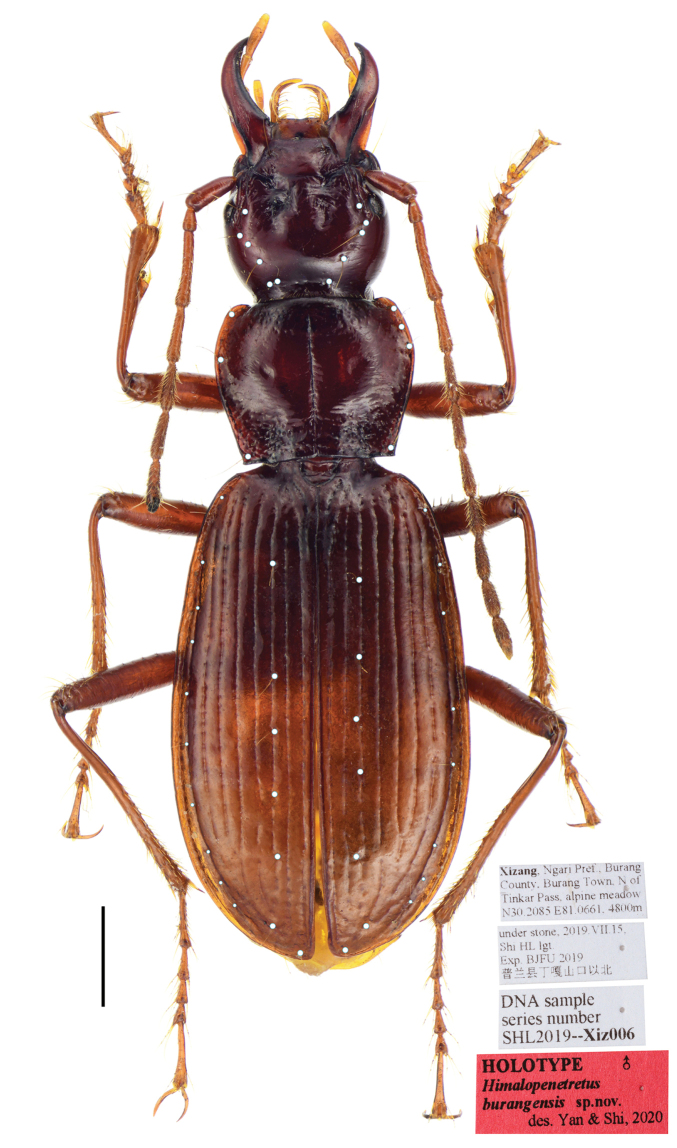
*Himalopenetretus burangensis* sp. nov. (holotype male, IZAS). Habitus and labels. Scale bar: 1.0 mm.

Habitus as in Figure 1. Medium-sized for a Patrobini species (L = 9.2 mm; EW = 2.9 mm).

*General appearance*: Dorsal side reddish brown, shiny, without metallic luster; head, mandibles and pronotum brown, elytra a little lighter; antennae, palpi and legs light reddish-brown; ventral side largely dark brown, abdominal sternum reddish-brown. Dorsal side glabrous and smooth except for lateral margins and basal foveae of pronotum punctate. Microsculpture invisible on head and pronotum, isodiametric on elytra, hardly visible near base, strong in apical third.

**Figures 2–7.**
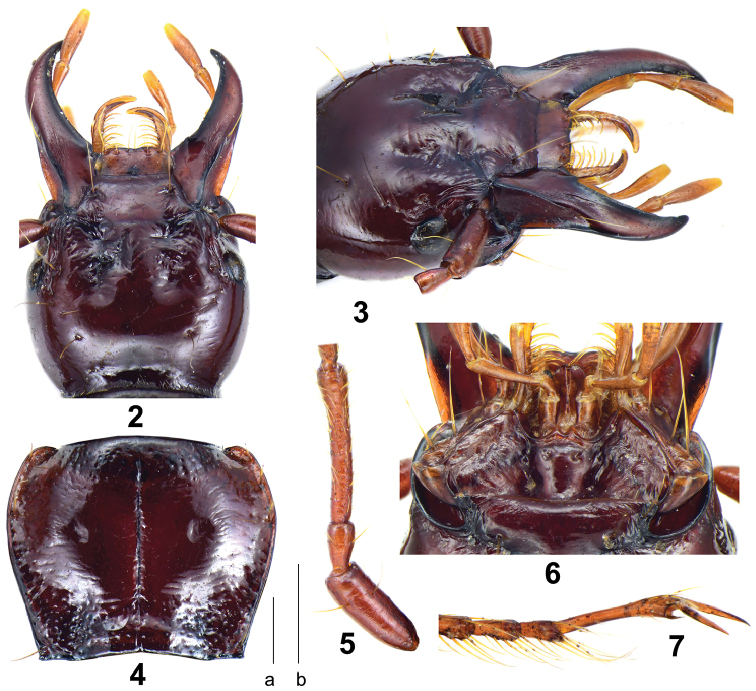
*Himalopenetretus burangensis* sp. nov. (holotype male, IZAS). **2** Dorsal side of head **3** Lateral side of head **4** Dorsal side of pronotum **5** First three segments of antennae **6** Labium **7** Lateral side of metatarsomeres. Scale bars: 0.5 mm (**a** for **2–4**, **b** for **5–7**).

*Head*: Broad and ovate; surface smooth, without prominent punctures. Mandibles strongly elongated, narrow, without an apical tooth (Figs 2, 3); apical maxillary palpomere broadest in middle, penultimate and antepenultimate ones with ring of apical setae; ligula with two apical setae. Antennae pubescent from third antennomere; first antennomere plurisetose, with one seta distinctly longer than the others; second antennomere glabrous except subapical ring of setae (Fig. 5). Eyes very small, inconspicuous, not convex; temporae long, much longer than eye diameter, moderately tumid; neck constriction rather shallow. Frontal furrows short but very deep, extended to level of posterior edge of eyes, slightly divergent posteriorly, almost parallel; with five or six setae between eyes and neck constriction on each side (holotype with six on the left side and five on the right side), including: one supraorbital seta near level of middle of eyes, three setae between posterior margins of eyes and neck constriction, one or two setae adjoining to neck constriction. Mentum basally with two rather deep longitudinal foveae, tooth narrow and bifid, apical notch shallow; submentum with two setae on each side (Fig. 6).

*Pronotum*: Nearly quadrate, a little broad, PW/PL = 1.24, faintly wider than head, PW/HW = 1.23, widest near anterior third, fairly convex, moderately constricted posteriorly. Anterior margin nearly straight; lateral margins fairly rounded in front, without prominent sinuation before posterior angles; posterior margin nearly straight; anterior angles rounded but distinct, slightly protruding anteriorly; posterior angles near rectangular, apex slightly pointed outwards. Anterior transverse impression shallow, sparsely punctate; basal foveae shallow, coarsely punctate and wrinkled; disk smooth, with a pair of shallow concavities to the side of median line (Fig. 4); median line distinct, almost reaching both extremities; lateral grooves coarsely punctate. Lateral margins each with two or three setae before middle (holotype with two setae on the left and three setae on the right); one pair of setae situated a little before posterior angles.

*Elytra*: Oblong-ovate, depressed; EL/EW = 1.66, widest near posterior third; humeri narrowly rounded, humeral tooth indistinct; lateral margins fairly widened and flat. Intervals slightly convex; striae well incised, finely punctate basally; scutellar stria long, situated between elytra suture and first striae, apex free, scutellar pore absent; third interval with five setiferous pores, all adjoining third stria, the first one near basal fifth; fifth interval with one setiferous pore near apex; umbilicate series on ninth interval composed of eight to ten pores, nearly equally arranged, a little denser in posterior areas.

*Ventral side*: Prosternum smooth, propleuron densely punctate; mesosternum and mesopleuron wrinkled, mesopleuron with sporadic coarse punctures; mesepimeron narrow, slightly widened laterally, suture separating mesepisternum and mesosternum joining lateral margin of metasternum; metepisternum rather long and narrow, not punctate. Lateral areas of abdominal sternites slightly rugose, abdominal sternite IV to VI with two setae near middle on each side; VII with one or two setae on each side in male (holotype with two setae on the left and one seta on the right).

*Legs*: Males with the first two protarsomeres slightly expanded, the second protarsomere distinctly wider than the third one which nearly triangular; the fourth protarsomere evidently bilobed; metatrochanter normal, not protruding or exceeding lateral margin of body; tarsomeres generally glabrous dorsally, only with a few very minute setae; the fifth meso- and metatarsomeres generally glabrous ventrally, with one to three pairs of minute setae (Fig. 7).

**Figures 8–13.**
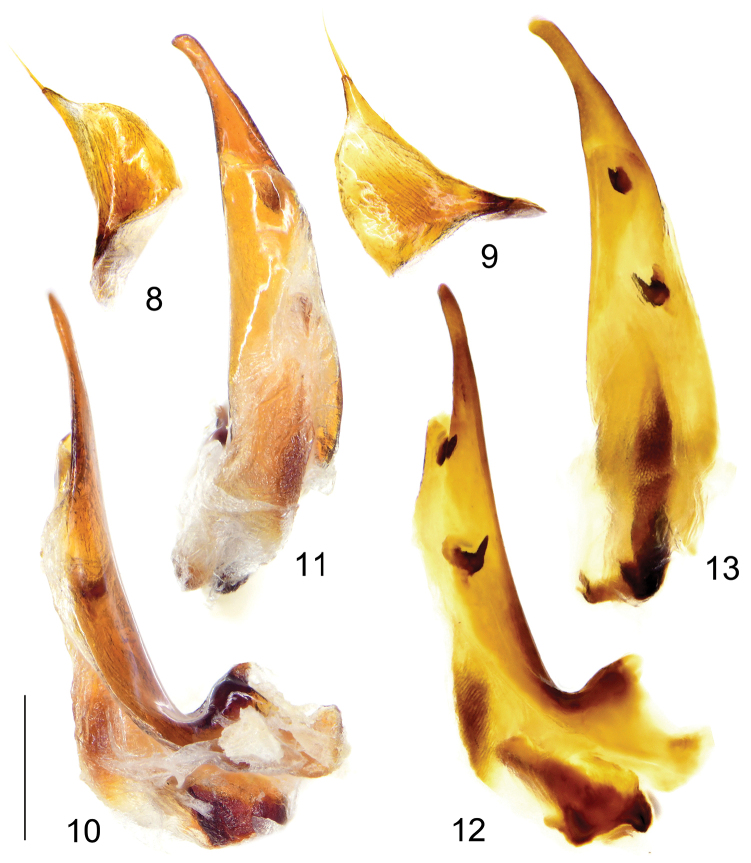
*Himalopenetretus burangensis* sp. nov. (holotype male, IZAS). **8** Right paramere of aedeagus **9** Left paramere of aedeagus **10–13** Median lobe of aedeagus, right lateral view (**10, 12**), ventral view (**11, 13**) **12, 13** were captured after the genitalia were treated with a 10% KOH solution for 12h to show features of the endophallus. Scale bar: 0.5 mm.

*Male genitalia* (Figs 8–13): Median lobe of aedeagus strongly bent at base, gutter-shaped and opened dorsally; in lateral view apical lamella nearly straight; in dorsal view apical lamella very elongate and narrow, gradually attenuated towards apex and prominently twisted leftwards, apex capitate, forming a faint hook to the left. Armature of endophallus consisting of two groups of copulatory pieces: proximal one near middle with sharp and hooked apex and a rudimentary flagellum; distal one near end of apical orifice, smaller and bilobed. Left paramere larger than right one, both short but sharply contracted towards apex, each with two long apical setae.

Female unknown.

#### Distribution.

**Map 1.**
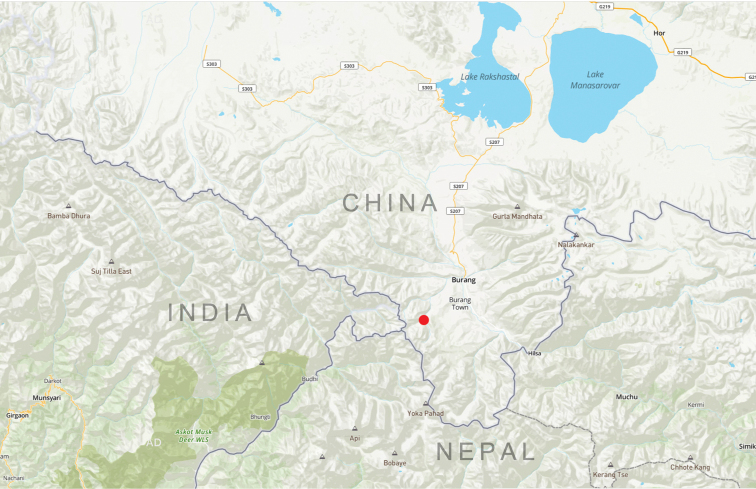
Distribution map for *Himalopenetretus burangensis* sp. nov. (red).

This species is known only from the holotype from Burang, Xizang (Map 1).

#### Etymology.

The new species is named after its type locality, Burang County.

#### Remarks.

Among the three species of the genus *Himalopenetretus*, *H. burangensis* sp. nov. could be closest to *H. franzi* for their near distribution ranges and morphological similarities. In particular, the dorsally glabrous tarsomeres of these two species could be of taxonomic significance.

The results of a phylogenetic analysis by Zamotajlov (2002) indicate a close relationship between *Himalopenetretus* and *Ledouxius*, and the subgenus Ledouxius (Hasarotretus) has some intermediate character states between these two genera (Zamotajlov and Sciaky 2006). In general, *H. burangensis* sp. nov. fits most of the generic characters of *Himalopenetretus* previously suggested (Zamotajlov 2002), but the lateral margins of pronotum with two or three setae before middle make the new species somewhat resemble the genus *Ledouxius*. However, from its general habitus, male genitalia, and many other respects, the new species clearly belongs to the genus *Himalopenetretus*. Thus, the generic diagnosis of this genus should be slightly modified to include the new species, as follows: pronotum lateral margins with two to five setae before middle.

**Map 2.**
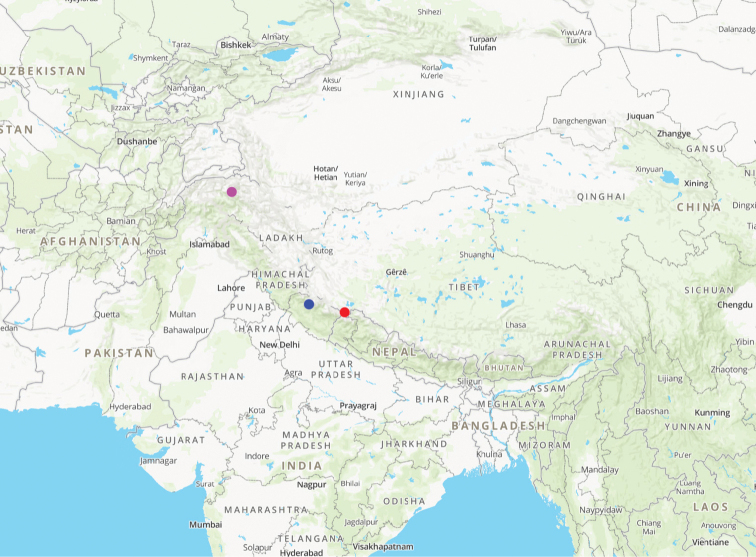
Distribution map for *Himalopenetretus* species: *H. burangensis* sp. nov. (red); *H. franzi* (blue); *H. falciger* (magenta).

The discovery of this new species extends the eastern limit of the known distribution of the genus *Himalopenetretus*. Considering that all the species of this genus are very rare, and that explorations in high mountains of the Western Himalayas are relatively inadequate, the discovery of additional new species is expected from the nearby areas, such as southwestern Xizang and Nepal (Map 2).

## Supplementary Material

XML Treatment for Himalopenetretus

XML Treatment for Himalopenetretus burangensis
